# Rare Variants and Polymorphisms of *FBN1* Gene May Increase the Risk of Non-Syndromic Aortic Dissection

**DOI:** 10.3389/fgene.2022.778806

**Published:** 2022-01-27

**Authors:** Meichen Pan, Lianjie Li, Zehao Li, Shu Chen, Zongzhe Li, Yuning Wang, Henghui He, Lihua Lin, Haihao Wang, Qian Liu

**Affiliations:** ^1^ Department of Forensic Medicine, Tongji Medical College of Huazhong University of Science and Technology, Wuhan, China; ^2^ Division of Thoracic Surgery, Union Hospital, Tongji Medical College of Huazhong University of Science and Technology, Wuhan, China; ^3^ Division of Cardiology, Departments of Internal Medicine and Genetic Diagnosis Center, Tongji Hospital, Tongji Medical College, Huazhong University of Science and Technology, Wuhan, China; ^4^ Division of Thoracic Surgery, Tongji Hospital, Tongji Medical College of Huazhong University of Science and Technology, Wuhan, China

**Keywords:** FBN1, cbEGF domains, cysteine residues, SNP, aortic dissection, splicing/truncation variants

## Abstract

Aortic dissection (AD) is a cardiovascular disease characterized by high mortality and poor prognosis. Although *FBN1* is associated with syndromic AD, its association with non-syndromic AD remains unclear. In this study, DNA samples from 90 Chinese individuals with non-syndromic AD (60 Stanford A, 30 Stanford B types) were analyzed to determine the relationship between diverse genotypes of the *FBN1* gene and non-syndromic AD. Eleven pathogenic/likely pathogenic variants (1 novel) were identified in 12.2% of patients with non-syndromic AD. Patients with positive variants suffered from AD at a younger age than those in the negative variant group. Among the six positive missense mutations associated with cysteine residue hosts, four (66.7%) were Stanford A AD, whereas two (33.3%) were Stanford B AD. Three (100%) positive splicing/truncation variant hosts were Stanford A AD. The splicing/truncation variants and missense variants involving cysteine residues in the *FBN1* gene increased the risk of Stanford A AD. Ten common SNPs that increased susceptibility to AD were identified. In particular, five SNPs were detected significantly in Stanford A AD, whereas another four SNPs were significantly detected in Stanford B AD. These significant variants can function as biomarkers for the identification of patients at risk for AD. Our findings have the potential to broaden the database of positive mutations and common SNPs of *FBN1* in non-syndromic AD among the Chinese population.

## Introduction

Aortic dissection (AD) is usually asymptomatic until the aorta ruptures and has a high risk of mortality and poor prognosis (Li et al., 2017). Presenting with overlapping clinical manifestations, AD can occur in individuals with genetic syndromic diseases, such as Marfan syndrome (MFS, OMIM 154700) or Ehlers-Danlos syndrome (EDS, OMIM 130050), causing vascular dysfunction (Regalado et al., 2016). However, a vast majority of AD cases (approximately 80%) usually occur in non-syndromic forms, such as familial thoracic AD (fTAAD, OMIM 607086) and sporadic AD (Guo et al., 2011; Hoffjan, 2012; Goldfinger et al., 2014). Over the last 2 decades, numerous genes (e.g., *FBN1*, *ACTA2*, *TGFBR1/2*, and *SMAD3*) have been identified as predisposing humans to syndromic AD and fTAAD (Hoffjan, 2012; Zhang and Wang, 2016; Pinard et al., 2019).

*FBN1* is a classic gene associated with MFS and encodes fibrillin-1 as a structural macromolecule that forms extracellular matrix (ECM) microfibrils (Sakai et al., 2016). Fibrillin microfibrils play many critical functional roles and serve as scaffolds for elastin deposition in the ECM (Fahrenbach et al., 1966). They are essential components for maintaining the structural integrity of both the aortic wall and the suspensory ligament of the eye lens and for regulating members of the transforming growth factor beta (TGFβ) family to sequester them to induce the formation and repair of organs (Sakai et al., 2016). Owing to these multiple functions, *FBN1* variants have been associated with pleiotropic clinical phenotypes. Moreover, some studies have suggested that variants in different *FBN1* regions are related to different phenotypes. However, the spectrum of known *FBN1* variants remains incomplete, and the association between *FBN1* and non-syndromic AD is unclear.

Based on the widely used Stanford system, dissections involving the ascending aorta are classified as Stanford A AD, and those involving only the descending aorta are classified as Stanford B AD (Gawinecka et al., 2017). Stanford A AD is a high-risk subtype and requires surgical intervention, with ∼50% of the patients succumbing to death before hospital admission, high mortality and morbidity in survivors after hospitalization, and emergency surgical repair (Howard et al., 2013). In addition, in our daily forensic practice, the majority of sudden death cases related to AD primarily include Stanford A AD. As some of the genes that increase the risk for AD have been identified, it is important to further investigate whether diverse genotypes of these genes may be linked to different types of AD (especially Stanford A AD), which would be useful in personalized preventive surgery. Therefore, samples from 90 Chinese Han individuals with non-syndromic AD were sequenced, not only to identify and report the frequency of *FBN1* pathogenic/likely pathogenic variants in AD, but also to determine the correlation between common single nucleotide polymorphisms (SNPs) and AD. Furthermore, we explored possible genotypic differences between the two AD types. We also investigated the presence of a hotspot mutation region, according to the distribution characteristics of the common SNPs and pathogenic/likely pathogenic variants identified along the gene.

## Materials and Methods

### Study Subjects

Ninety individuals with non-syndromic AD were recruited from the Han Chinese population with non-syndromic AD. The diagnosis criteria of AD were confirmed using the patient medical history, computed tomography (CT) or magnetic resonance imaging (MRI), or pathological evidence of AD from postmortem autopsy. Patients with syndromic AD (such as MFS, EDS, Loeys-Dietz syndrome), aneurysm only, or traumatic AD, as well as those not of Han Chinese ethnicity were excluded. Peripheral blood was obtained from 79 in-hospital patients diagnosed with AD at several hospitals in Wuhan, China, between January 2016 and December 2020. Heart arterial blood was also collected from 11 patients who died from AD based on a precise diagnosis at the Department of Forensic Medicine, Tongji Medical College, Huazhong University of Science and Technology, Wuhan, China, from January 2016 to December 2020. Data were also collected from the medical and anatomical records. The control group consisted of 568 unrelated healthy (with no AD) Han Chinese ethnicity individuals from the Novo-Zhonghua project.

All sample collection procedures met the ethical guidelines of Tongji Medical College, Huazhong University of Science and Technology (approval number [2017] IEC (S059)). Consent forms were signed by the patients or legal relatives of the deceased.

### Determination of Genotypes

Peripheral or heart blood samples extracted from each subject were stored in ethylenediaminetetraacetic acid (EDTA) tubes. Genomic DNA was extracted using a TIANamp Blood DNA Kit (TIANGEN, DP318). The *FBN1* sequencing data of 90 individuals were obtained by next-generation sequencing on the Illumina HiSeq 4,000 platform. The coverage obtained was 50% on average, at a depth of 100×.

The obtained sequencing data were compared with the human reference genome (GRCh37/hg19) to sort out variants from the raw sequences, and the qualified variants were further annotated using ANNOVAR (Wang et al., 2010). Variants with a minor allele frequency (MAF) < 0.01 in all three databases (1000Genomes, ExAC, and ESP6500) were defined as rare variants. Variants were annotated and classified into five categories (pathogenic, likely pathogenic, uncertain significance, likely benign, and benign) according to the American College of Medical Genetics (ACMG) Guidelines Revisions (Richards et al., 2015). Both pathogenic and likely pathogenic variants were defined as positive variants. Variants with an MAF >0.05, in the 1,000 Genomes Project, ExAC, and ESP databases, were defined as common SNPs and were selected for analysis of associations within different phenotypes of AD.

### Statistical Analysis

Continuous variables are presented as mean ± standard deviation, and all comparisons of mean values were performed using unpaired *t*-test or analysis of variance (ANOVA). Categorical data are presented as percentages or rates and were compared using the chi-square test or two-tailed Fisher’s exact test. Odds ratios (ORs) and associated 95% confidence intervals (CIs) were used to evaluate the association of each identified *FBN1* common SNPs with AD susceptibility. A significant P-value corrected by the Bonferroni correction was used owing to multiple comparisons of the *FBN1* common SNPs studied. Hardy–Weinberg equilibrium was evaluated using PowerStats v1.2 software to examine whether or not the samples showed group representation. Other statistical analyses were performed using SPSS version 25.0. All tests were two-sided and assessed at a significance level of *p* < 0.05.

## Results

### Clinical Characteristics

The study group consisted of 90 individuals diagnosed with non-syndromic AD. The demographic and clinical features of the study participants were summarized in Table 1. The mean age for the onset of AD was 48.76 ± 10.20 years, ranging from 21 to 72 years. Of these individuals, 68.9% (62/90) were males and 31.1% (28/90) were females; 60 patients (66.7%) had dissection involving the ascending aorta (Stanford A AD) and 30 (33.3%) had dissection involving only the descending aorta (Stanford B AD). In addition, 51 individuals (56.7%) presented with hypertension, 23 (25.6%) had a history of smoking, 14 (15.6%) had coronary atherosclerotic heart disease, and 8 (8.9%) presented with coronary artery dissection. The specific clinical and demographic information of the patients is presented in Supplementary Table S1.

**TABLE 1:** The demographic and clinical features of study participants.

Characteristics	Stanford A AD (*n = 60)*	Stanford B AD (*n* = 30)	Total AD (*n* = 90)
Age, years	25/66/47.43	21/72/51.43	21/72/48.76
*Min/max/average*			
Gender	42 (70.0%)	20 (66.7%)	62 (68.9%)
*Male, n (%)*			
Hypertension *n (%)*	29 (48.3%)	22 (73.3%)	51 (56.7%)
Smoking *n (%)*	18 (30.0%)	5 (16.7%)	23 (25.6%)
Coronary artery dissection *n (%)*	7 (11.7%)	1 (3.3%)	8 (8.9%)
Coronary atherosclerotic heart disease *n (%)*	12 (20.0%)	2 (6.67%)	14 (15.6%)

### General Information of Variants

A total of 125 variants of *FBN1* were identified from the 90 individuals with AD, including 99 variants in introns, 22 variants in exons, two splicing variants, and two variants in the 5’ UTR area. The distribution of genotypes was consistent with Hardy–Weinberg equilibrium (*p* > 0.12) in the 90 AD patients included in the study.

There were 44 rare variants (MAF <0.01). Among these, five pathogenic and six likely pathogenic variants (for a total of 11 positive variants) were identified. All 11 positive variants were heterozygous and absent in the healthy control group. Detailed genetic information of these 11 positive variants was provided in Table 2. Among these, 10 positive variants were reported to be associated with AD in the ClinVar database or HMGD, and one was novel (not previously reported), suggesting that similar pathogenic mechanisms are shared among different types of AD. The genetic diagnostic yield of *FBN1* positive variants was 12.2% (11/90) in the experimental group, 11.7% (7/60) in Stanford A AD, and 13.3% (4/30) in Stanford B AD, with no significant difference, according to type.

**TABLE 2:** Detailed genetic and clinical information of AD patients harboring positive variants in FBN1.

Sample, stanford type	Age ranges, sex	Position	Function	Domain	Nucleotide change	Amino change	Functional predicted results in sift, mutation taster	Classification	Novel	In control group	ACMG criterion fulfilled
A59, Stanford A	40–45, M	chr15: 48,714,188	missense	cbEGF-like#39	7531T > C	p.Cys2511Arg	D, D	Pathogenic	Reported	Absent	PS1+PM1+PM2+PM5+PP2+PP3+PP5
C2, Stanford A	40–45, F	chr15: 48,795,983	splicing	/	2,113+1G > A	/	/, D	Pathogenic	Reported	Absent	PSV1+PM2+PP3+PP5
C13, Stanford A	30–34, F	chr15: 48,717,610	missense	cbEGF-like#38	c.7409G > A	p.Cys2470Tyr	D, D	Likely Pathogenic	Reported	Absent	PM1+PM2+PM5+PP2+PP3+PP5
C21, Stanford B	35–40, F	chr15: 48,760,243	missense	TGFBP#4	c.4639A > G	p.Thr1547Ala	T, D	Pathogenic	Reported	Absent	PM1+PM2+PP2+PP3
C24, Stanford A	40–45, F	chr15: 48,776,059	frameshift deletion	cbEGF-like#16	c.3793delT	p.Cys1265fs	/,/	Likely Pathogenic	Reported	Absent	PSV1+PM1+PM2+PP3
C38, Stanford A	30–35, M	chr15: 48,800,791	missense	cbEGF-like#5	c.1825C > T	p.Arg609Cys	D, D	Pathogenic	Reported	Absent	PM1+PM2+PP2+PP3+PP5
C46, Stanford A	46–50, F	chr15: 48,722,988	missense	cbEGF-like#35	c.6751T > C	p.Cys2251Arg	D, D	Likely Pathogenic	Reported	Absent	PS1+PM1+PM2+PM5+PP2+PP3+PP5
C52, Stanford B	35–40, M	chr15: 48,797,266	missense	cbEGF-like#6	c.1916G > C	p.Cys639Ser	D, D	Likely Pathogenic	Reported	Absent	PS1+PM1+PM2+PM5+PP2+PP3+PP5
C61, Stanford B	46–50, F	chr15: 48,766,769	missense	cbEGF-like#18	c.4043G > A	p.Cys1348Tyr	D, D	Pathogenic	Reported	Absent	PM1+PM2+PM5+PP2+PP3+PP5
C87, Stanford A	40–45, M	chr15: 48,758,018	frameshift deletion	TGFBP#4	c.4784delT	p.Phe1595fs	/,/	Likely Pathogenic	Novel	Absent	PVS1+PM1+PM2
C105, Stanford B	56–60, M	chr15: 48,722,942	missense	cbEGF-like#35	c.A6797G	p.Lys2266Arg	D, D	Likely Pathogenic	Reported	Absent	PM1+PM2+PP2+PP3

AbbreviationsM, male; F, female; chr, chromosome; cbEGF, calcium-binding epidermal growth factor-like domain; TGFBP, transforming growth factor β-binding protein-like domains; D, deleterious; T, tolerated.

### Details of the 11 Pathogenic/Likely Pathogenic Variants

All the 11 positive variants were unique to a single patient; that is, the same pathogenic or likely pathogenic variants were never linked to more than one individual in our study. Of these, eight (72.7%) were missense variants, two were frameshift deletions, and one was a splicing variant. Six of the eight missense variants (75%) involved cysteine residues: five unique missense variants involved substitution of a cysteine, and one missense variant created a new cysteine (p.Cys2511Arg in A59, p.Cys2470Try in C13, p.Arg609Cys in C38, p.Cys2251Arg in C46, p.Cys639Ser in C52, and p.Cys1348Try in C61). Specifically, in Stanford A AD, there were four missense variants, two frameshifts, and one splicing variant, whereas in Stanford B AD, there were four missense variants. Interestingly, four missense variants in Stanford A AD were all linked to cysteine residues, whereas only 2/4 missense variants in Stanford B AD were related to cysteine residues.

Variants in the 5′ region (exons 1–10), 3′ region (exons 59–65), and middle region (exons 24–32) of the *FBN1* gene are related to specific manifestations (Sakai et al., 2016). In the AD group, only two positive variants were detected in the 3′ region, one in the middle region, and there was no 5′ region-positive variant. The above three positive variants were found in Stanford A AD.

**FIGURE 1:**
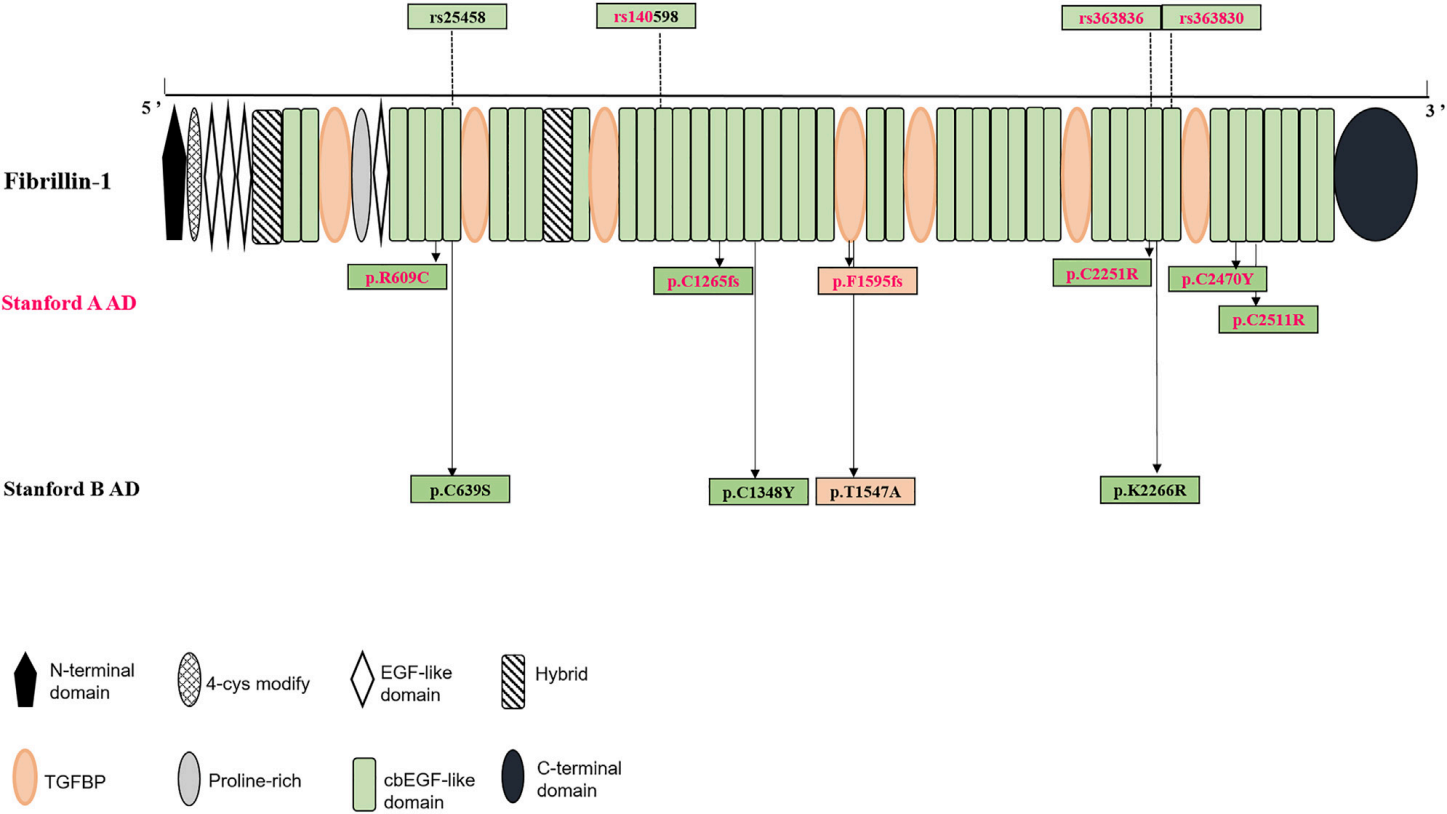
Structural domain of fibrillin-1 (encoded by FBN1 gene) and distribution of both positive variants and significant SNPs in the protein were shown.

The calcium-binding epidermal growth factor-like (cbEGF-like) domains are critical functional areas in fibrillin structures (http://www.umd.be/FBN1/). Among the 11 positive variants of the *FBN1* gene, eight (seven missense variants and one frameshift deletion) were detected in this module, that is, the positive variants in the cbEGF domains were clearly dominant in our study group (Figure 1). The remaining three positive variants were located in the TGFβ-binding-protein-like (TGFBP) domains, homologous with TGFβ-1 binding protein, which were mainly interspersed among the cbEGF domains. Specifically, 8 AD individuals showed positive variants in the cbEGF domains, with 5/8 (62.5%) of these individuals diagnosed with Stanford A AD. Moreover, three patients had TGFBP domain-positive variants, two of which (66.7%) were diagnosed with Stanford A AD.

### Association of Common SNPs With AD

Associations of common SNPs with AD susceptibility were evaluated using the chi-square test, which provided ORs, 95% CIs, and level of significance (P). The MAFs of 10 SNPs were significantly higher in the AD group than in the healthy control subjects, which may appear to be significantly associated with an increased risk of AD. Detailed information on MAFs and OR values of these 10 significant SNPs is shown in Table 3. Among these, 4 SNPs (rs140598, rs363830, rs25458, and rs363836) were in the exon, while the rest 6 SNPs (rs11853943, rs140605, rs142758852, rs363838, rs55840194, and rs57512865) were in the intron. Notably, these four exonic SNPs were all located in the cbEGF domains (Figure 1), which further confirmed the importance of the cbEGF domains of the *FBN1* and AD groups.

**TABLE 3:** 10 significant SNPs between AD group and reference group.

SNPs (locations)	AD group (*n* = 90)	Reference group (*n* = 568) MAF
	MAF, OR (CI, 95%), P value	
rs140598 (exon/cbEGF#13)	0.42, 2.56 (1.61–4.07), *p* = 0.000044	0.22
rs363830 (exon/cbEGF#36)	0.19, 3.07 (1.66–5.70), *p* = 0.000205	0.07
rs25458 (exon/cbEGF#06)	0.46, 1.80 (1.15–2.83), *p* = 0.01	0.32
rs363836 (exon/cbEGF#35)	0.19, 2.99 (1.62–5.54), *p* = 0.000285	0.07
rs11853943 (intron)	0.47, 1.87 (1.19–2.94), *p* = 0.006	0.32
rs140605 (intron)	0.46, 1.79 (1.14–2.81), *p* = 0.01	0.32
rs142758852 (intron)	0.19, 2.58 (1.41–4.73), *p* = 0.002	0.08
rs363838 (intron)	0.19, 3.07 (1.66–5.70), *p* = 0.000205	0.07
rs55840194 (intron)	0.19, 3.00 (1.62–5.54), *p* = 0.000285	0.07
rs57512865 (intron)	0.31, 1.69 (1.03–2.76), *p* = 0.03	0.21

AD, aortic dissection; MAF, minor allele frequency; OR, odds ratio; CI, confidence interval.

All 10 significant SNPs showed different distributions between Stanford A AD and Stanford B AD. The detailed information is presented in Table 4. Clearly, MAFs of five SNPs (rs363830, rs363836, rs142758852, rs363838, and rs55840194) were significantly higher in Stanford A AD than control group, but not in Stanford B AD. That is, these five SNPs may increase the susceptibility to Stanford A AD. Simultaneously, another four SNPs (rs25458, rs11853943, rs140605, and rs57512865) were more relevant to Stanford B AD. The remaining 1 SNP (rs140598) was significant in both Stanford A AD and Stanford B AD.

**TABLE 4:** Detailed distribution of 10 significant SNPs in Stanford A AD and Stanford B AD.

SNPs	Stanford A AD group	Stanford B AD group	Reference group MAF
	MAF, OR (CI,95%), P value	MAF, OR (CI,95%), P value	
rs363830 (exon)	0.20, 3.30 (1.62–6.71), *p* = 0.001	0.17, No significance	0.07
rs363836 (exon)	0.20, 3.21 (1.58–6.52), *p* = 0.001	0.17, No significance	0.07
rs142758852 (intron)	0.20, 2.78 (1.38–5.58), *p* = 0.003	0.17, No significance	0.08
rs363838 (intron)	0.20, 3.30 (1.62–6.71), *p* = 0.001	0.17, No significance	0.07
rs55840194 (intron)	0.20, 3.21 (1.58–6.52), *p* = 0.001	0.17, No significance	0.07
rs25458 (exon)	0.38, No significance	0.60, 3.23 (1.53–6.86), *p* = 0.001	0.32
rs11853943 (intron)	0.38, No significance	0.63, 3.70 (1.72–7.92), *p* = 0.000370	0.32
rs140605 (intron)	0.38, No significance	0.60, 3.21 (1.51–6.80), *p* = 0.001	0.32
rs57512865 (intron)	0.23, No significance	0.47, 3.27 (1.55–6.88), *p* = 0.001	0.21
rs140598 (exon)	0.40, 2.34 (1.35–4.07), *p* = 0.002	0.47, 3.07 (1.46–6.46), *p* = 0.002	0.22

AD, aortic dissection; MAF, minor allele frequency; OR, odds ratio; CI, confidence interval. Bonferroni-corrected significance threshold *p* < 0.025.

Among the four exonic SNPs, two were significantly associated with higher MAFs in Stanford A AD, 1 was significant in Stanford B AD, and 1 was significant with higher MAFs in both Stanford A AD and Stanford B AD (Table 4).

### Correlation Between Genotype and Phenotype

**FIGURE 2:**
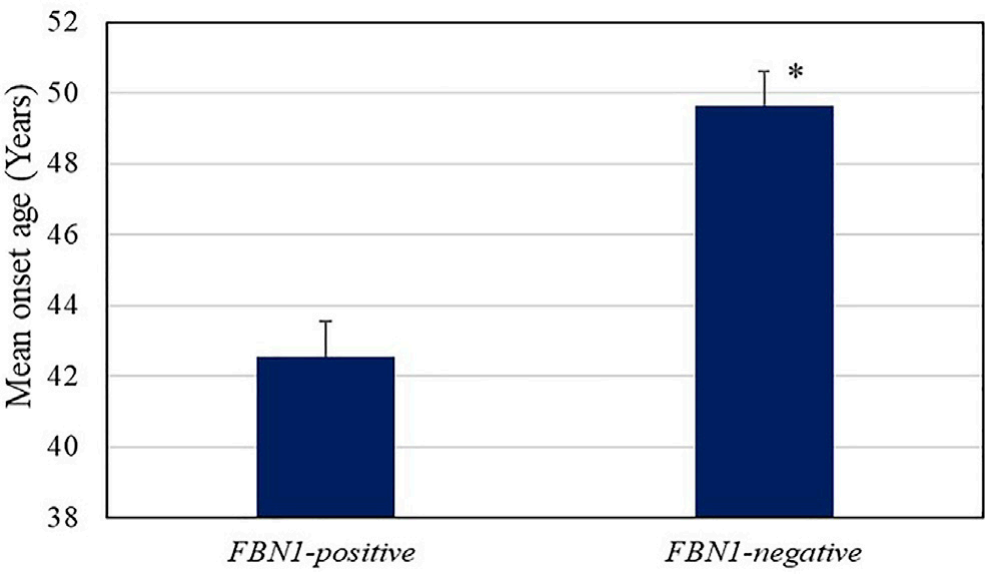
Age of onset of AD in the *FBN1* positive variant (42.55 ± 6.95 years) and negative groups (49.63 ± 10.31 years, *p* = 0.03). The error bars indicate standard error.

The mean onset age for AD in individuals with *FBN1* positive-variant (42.55 ± 6.95 years) was much lower than that of the non-*FBN1* positive-variant group (49.63 ± 10.31 years, *p* = 0.03, Figure 2), whereas the mean onset age in the significant SNPs group showed no differences from that of other groups. The fact that AD patients with *FBN1* positive-variant tended to suffer from AD at an earlier age supports the pathogenicity of these *FBN1* variants. In addition, compared to the *FBN1* negative-variant group, the missense variant-positive group showed a smaller mean onset age (42.00 *vs*. 49.63 years, *P*<0.05), while the mean onset age in the truncating and splicing group (44.00 years) was not significantly different from that of the *FBN1* negative-variant group. Further, the mean onset ages in the cbEGF (42.88 years) and TGFBP domain (41.00 years) group showed no differences with those in the *FBN1* negative-variant group, and there were no differences in those between the two domain groups.

In contrast to the male predominance in the *FBN1* negative-variant group (72.2%, 57/79), females were predominant in the *FBN1* positive-variant group (54.6%, 6/11), with no statistical differences. Moreover, the *FBN1* positive-variant group showed a tendency for a less frequent history of hypertension (45.5 *vs*. 58.2%) and smoking (9.1 *vs*. 27.8%). The precise genetic information for Stanford A and Stanford B AD is provided in Table 5.

**TABLE 5:** Specific positive variants and SNPs information of *FBN1* between Stanford A and Stanford B cases.

Classification	Stanford type AD	
	Stanford A	Stanford B
*FBN1* positive variants hosts	7	4
Missense	4	4
Cysteine residues involved	4	2
Frameshift deletion/splicing	3	0
3′ region positive variants	2	0
5′ region positive variants	0	0
Positive variants in other region	5	4
CbEGF domains	5	3
TGFBP domains involved	1	1
Significant exonic SNPs of *FBN1*	3	2
CbEGF domains	3	2

## Discussion

### Positive Variant Rate of *FBN1*

*FBN1* was initially reported to be associated with MFS (Comeglio et al., 2007; Faivre et al., 2007). However, further research has revealed that many other clinical phenotypes without syndromic disease are associated with *FBN1*. Lesauskaite et al. (Lesauskaite et al., 2015) suggested that five *FBN1* polymorphisms increased susceptibility to AD. Using exome sequencing, Regalado et al. (Regalado et al., 2016) found that the frequency of *FBN1* mutations was 2.7% (5/183) in patients with familial thoracic aortic aneurysms and dissection. In addition, Tan et al. (Tan et al., 2017) detected a total of 26 *FBN1* positive variant cases among 687 sporadic AD cases, with a detection rate of 3.9%. In our study, 11 *FBN1* positive-variants (1 of which were novel) were identified, with a genetic diagnostic yield of 12.2% (11/90). Collectively, these data emphasize the pathogenicity of *FBN1* in individuals with AD, and the clinical differences between *FBN1* positive-variant carriers and non-*FBN1* positive-variant (*FBN1* negative-variant) carriers were also confirmed. Moreover, the *FBN1* positive-variant group show a lower frequency of history of hypertension and smoking than *FBN1* negative-variant group, which are well-known risk factors for AD.

Notably, the positive variant rate of *FBN1* was high in our cohort. This wide range may be attributed to differences in the ethnicity of the patients in different studies. Although the population in our study was the same as that in the study by Tan et al. (Tan et al., 2017), there was a difference in the diagnostic yield of *FBN1*. One possible reason is that the proportion of AD type differed between our study group and that of Tan et al. (Tan et al., 2017). Our study group mainly comprised patients with the more severe type of AD, Stanford A (66.7%), whereas only 23.9% of the cohort of the previous study had Stanford A AD. However, further investigations are needed to confirm the factors contributing to the differences in these rates.

### Different Types in Positive Variant of *FBN1* Types

Different types of *FBN1* variants have been reported in various studies (Robinson et al., 2006). However, missense variants have been reported to account for approximately 2/3 of MFS cases (Sakai et al., 2016), commonly involving the substitution or creation of new cysteine residues (Arbustini et al., 2005; Rommel et al., 2005). Positive variants involving cysteine residues may disturb right intra-domain disulfide bond formation, ultimately resulting in the alteration of the fibrillin-1 structure in the medial layer of the aorta (Tan et al., 2017). Among the 11 *FBN1* positive variants, the large proportion of cysteine residues in our identified variants as well as the younger mean onset age of the group corroborates this point. In our study, positive missense variants (that is, missense variants of 11 *FBN1* positive variants) accounted for a larger proportion (8/11, 72.7%). Moreover, in line with previous studies, missense variants involving cysteine accounted for a large proportion of these variants. It is worth noting that four missense variants in Stanford A AD were all associated with cysteine residues, while only 2/4 missense variants in Stanford B AD that were related to cysteine residues, suggesting the need to pay more attention to positive missense variants involved in cysteine residues in Stanford A AD.

Meanwhile, some studies (Wang et al., 2003; Yu et al., 2006; Jin et al., 2007; Li et al., 2008; Zhao et al., 2009; Wang et al., 2013) found that positive truncating or splicing variants were significantly increased over positive missense variants in Chinese patients with cardiovascular defects. In addition, some studies reported that MFS patients with aortic events had a higher frequency of *FBN1* truncating (frameshift and nonsense) or splicing variants than those with no aortic events (Baudhuin et al., 2015), but this was not observed in our AD group. However, all three AD cases presented with splicing or truncation variants, which were all Stanford AD. In summary, among the six missense variants involved in cysteine residue hosts, four (66.7%) were cases of Stanford A AD, whereas 2 (3.3% (2/6) were Stanford B AD. Among the three splice or truncation variant hosts, 100% were Stanford A AD. Therefore, missense variants involved in cysteine residues and splicing/truncation variant hosts are more likely to suffer from a severe type of AD (Stanford A AD). Certain variant types and their affected clinical features in the Chinese population require further investigation and more data accumulation.

### Significant SNPs in Stanford A and Stanford B AD

Many studies have focused on the positive variants between the *FBN1* gene and AD. Nevertheless, the association between *FBN1* polymorphisms and AD warrants further investigation. For instance, Lesauskaite et al. (Lesauskaite et al., 2015) indicated that two SNPs of *FBN1* (rs1036477, rs2118181) were prone to increase the risk of ascending aortic aneurysm. In this study, a total of 10 *FBN1* SNPs were detected to be significant in the AD group: having five SNPs may increase susceptibility to Stanford A AD, and having 4 SNPs were linked to the risk of Stanford B AD.

### Specific Regions and Domains of the Identified Positive Variants

Specific regions of *FBN1* are associated with concrete phenotypes. Positive variants in the 5 region (exons 1–10) and 3′ region (exons 59–65) are more likely to have milder cardiovascular features (Robinson et al., 2002; Comeglio et al., 2007). Two positive variants in the 3 region were identified: c.G7409A leading to Cys2470Try in exon 60 from a 34-year-old female patient with Stanford A AD and c.T7531C leading to Cys2511Arg in exon 61 from a 40-year-old male patient with Stanford A AD. In addition, positive variants identified in the middle region of *FBN1* (exons 24–32), containing consecutive cbEGF-like domains, were linked to “neonatal MFS” and severe forms of MFS (Faivre et al., 2007; Faivre et al., 2009). One positive frameshift deletion variant was detected, c.3793delT, leading to Cys1265fs in exon 31, from a 45-year-old female patient with Stanford A AD. A few variants were concentrated in the above regions, and the remaining seven positive variants were evenly distributed in other *FBN1* areas without a hotspot or specific region. This information indicates that all the regions of *FBN1* are critical contributors to non-syndromic AD in our study.

### Positive Variants in cbEGF Domains and TGFBP Domains

Multiple functional domains constitute the fibrillin protein, including 47 epidermal growth factor-like (EGF-like) domains, 43 of which are cbEGF-like domains, seven are TGBP domains, two are hybrid modules, one is a proline-rich domain, one is a unique amino-terminal, and one is a terminal region (Wang et al., 2013) (Figure 1). Therefore, it would be useful to determine whether variants in different domains are associated with specific phenotypes.

In our study group, common SNPs showed a clear distribution along the fibrillin protein domains. Four exon SNPs were located in the cbEGF domains, 3/4 were significant in Stanford A AD, and 2/4 were significant in Stanford B AD. Among the 11 positive variants, eight were located in the cbEGF domains, and two were located in the TGFBP domains. Specifically, among the seven Stanford A AD positive variants, five were located in the cbEGF domain and one was in the TGFBP domain. Similarly, four positive variants of Stanford B AD, three were in the cbEGF domain and one in the TGFBP domain.

In addition, the structure of a four–domain fibrillin fragment (EGF2-EGF3-Hyb1-cbEGF1) and C-terminal LTBP1 fragment demonstrated a bipartite interaction, which presumably facilitated a force-induced/traction-based TGF-β activation mechanism (https://www.ncbi.nlm.nih.gov/Structure/pdb/5MS9). Therefore, variants in this region may be related to AD, and should be considered. While there were no notable positive variants in this region, we will focus on it in future studies.

There were certain limitations to our study. First, only variants of *FBN1* in patients with AD were analyzed. Second, the number of patients with AD enrolled in our study was not as large as that in other studies. Therefore, larger-scale testing should be carried out in the future to verify our findings and further confirm the distribution of the differences between Stanford A and Stanford B AD.

This comprehensive systematic study used *FBN1* sequencing information to assess the role of both common SNPs and positive variants (pathogenic/likely pathogenic variants) of AD risk in the Han Chinese population. Eleven positive variants (1 novel) were detected in 12.2% (11/90) of AD individuals. *FBN1* positive variants also led to an earlier onset of AD. Positive variants were spread along *FBN1* without concentrated or hotspot regions. However, it is noteworthy that both missense variants involved in cysteine residues and splicing/truncation variant hosts were more likely to suffer from a severe type of AD (Stanford A AD). Thus, the critical role of the variant type of *FBN1* was emphasized in this study, indicating that individuals with missense variants involved in cysteine residues and splicing/truncation variants may have a higher risk of developing Stanford A AD. In addition, 10 SNPs increased susceptibility to AD, which might be considered biomarkers for identifying individuals at risk for AD. In particular, five SNPs were detected significantly in Stanford A AD, while four SNPs were identified to be linked to Stanford B AD. Our study thus broadens the molecular pathology and database of common SNPs of *FBN1* associated with non-syndromic AD among the Chinese population.

Among the 7 positive FBN1 variants in Stanford A AD, 5 were found in cbEGF domains, 1 was in TGEFB domains, and the rest 1 was splicing. Among the 4 positive FBN1 variants in Stanford B AD, 3 were found in cbEGF domains while 1 was in TGEFB domains. Four significant exonic SNPs were identified in AD, of which 2/4 (rs363836, rs363830) were link to Stanford A AD, 1/4 (rs25458) was prone to Stanford B AD, and the remaining 1 (rs140598) was associated in both Stanford A AD and Stanford B AD. These 4 SNPs were all located in the cbEGF domains, as well.

## Data Availability

The data that support the findings of this study have been deposited into CNGB Sequence Archive (CNSA) of China National GeneBank DataBase (CNGBdb), https://db.cngb.org/cnsa/, with accession number CNP0002539.
