# Supplementary material for: Rare Variants and Polymorphisms of FBN1 Gene May Increase the Risk of Non-Syndromic Aortic Dissection
Source: Front Genet. 2022 Jan 27;13:778806. doi: 10.3389/fgene.2022.778806 (PMC8829505; doi:10.3389/fgene.2022.778806)
Supplement: Supplementary file 1 [file Table1.DOCX]

**Supplementary Table 1. The specific detailed clinical features in 90 study group.**

| **Case number** | **Stanford classification** | **Gender, Age range** | **Previous history** | **Other CVS involvement** | **Type of surgery** | **Outcomes** |  |  |  |  |  |
| --- | --- | --- | --- | --- | --- | --- | --- | --- | --- | --- | --- |
| A-1 | Type A | M，50-55 | H+S | HC+CAHD | / | Dead |  |  |  |  |  |
| A-16 | Type A | M，40-45 | / | CAHD | / | Dead |  |  |  |  |  |
| A-41 | Type B | M，50-55 | H | CAHD | / | Dead |  |  |  |  |  |
| A-44 | Type A | F，36-40 | / | HC+CAHD | / | Dead |  |  |  |  |  |
| A-54 | Type A | M，50-55 | / | CAHD | / | Dead |  |  |  |  |  |
| A-55 | Type A | F，50-55 | H | HC+CAHD | / | Dead |  |  |  |  |  |
| A-58 | Type A | M，30-35 | / | HC+CAHD | / | Dead |  |  |  |  |  |
| A-59 | Type A | M，40-45 | H | CAHD | / | Dead |  |  |  |  |  |
| A-74 | Type A | M，60-65 | / | HC+CAHD | / | Dead |  |  |  |  |  |
| A-81 | Type A | M，30-35 | S | HC+CAHD | / | Dead |  |  |  |  |  |
| A-86 | Type A | M，50-55 | H | CAHD | / | Dead |  |  |  |  |  |
| C-2 | Type A | F，40-45 | / | AR(1) | BT+TAR+SET | Postoperative and discharge |  |  |  |  |  |
| C-3 | Type A | M，46-50 | H | BT+LCCA+LSA+PCE+AR(1) | NCSA+AAR+TAR+SET | Postoperative and discharge |  |  |  |  |  |
| C-5 | Type A | F，40-45 | H | BT+PCE+AR(2) | AAR+TAR+SET | Postoperative and discharge |  |  |  |  |  |
| C-6 | Type A | M，46-50 | H | BT+LCCA+LSA+PCE | NCSA+AAR+TAR+SET | Postoperative and discharge |  |  |  |  |  |
| C-7 | Type A | M，46-50 | / | BT+PCE | CSA+AAR+TAR+SET | Postoperative and discharge |  |  |  |  |  |
| C-9 | Type B | M，36-40 | H+S | PCE+CAHD | AAR+TAR+SET+CABG | Postoperative and discharge |  |  |  |  |  |
| C-10 | Type A | F，66-70 | H | / | / | Patient rejected surgery and discharged |  |  |  |  |  |
| C-11 | Type A | F，50-55 | H | BT+LCCA+LSA | CSA+AAR+TAR+SET | Postoperative and discharge |  |  |  |  |  |
| C-13 | Type A | F，30-35 | / | BT+LCCA+LSA+HCA+RCA | BT+TAR+SET | Postoperative and discharge |  |  |  |  |  |
| C-14 | Type A | M，50-55 | S | BT+LCCA+LSA+LCA+PCE+AR(2)+MR（1） | BT+TAR+SET | Postoperative and discharge |  |  |  |  |  |
| C-15 | Type A | F，40-45 | / | BT+LCCA+LSA+LCA+RCA+PCE+AR（2） | BT+TAR+SET | Postoperative and discharge |  |  |  |  |  |
| C-16 | Type A | M，36-40 | H | PCE+AR(3) | / | Dead before surgery |  |  |  |  |  |
| C-18 | Type A | M，26-30 | S | BT+LCCA+LSA+LCA+RCA+PCE | CSA+AAR+TAR+SET | Postoperative and discharge |  |  |  |  |  |
| C-19 | Type B | M，46-50 | H | / | TAMTI | Postoperative and discharge |  |  |  |  |  |
| C-20 | Type B | M，50-55 | H+S | / | TAMTI | Postoperative and discharge |  |  |  |  |  |
| C-21 | Type B | F，36-40 | / | / | TAMTI | Postoperative and discharge |  |  |  |  |  |
| C-22 | Type A | M，40-45 | / | LCA+RCA+AR(3) | BT+TAR+SET | Postoperative and discharge |  |  |  |  |  |
| C-24 | Type A | F，46-50 | / | AR(3)+MR(1)+TR(2) | BT+TAR+SET | Postoperative and discharge |  |  |  |  |  |
| C-25 | Type A | M，46-50 | H+S | BT+LSA+LCA+AR(2) | LCSA+AAR+TAR+SET | Postoperative and discharge |  |  |  |  |  |
| C-28 | Type A | F，40-45 | H | BT+PCE | AAR+TAR+SET | Postoperative and discharge |  |  |  |  |  |
| C-29 | Type A | F，66-70 | / | BT+PCE | AAR+TAR+SET | Postoperative and discharge |  |  |  |  |  |
| C-30 | Type A | F，50-55 | H | PCE+AR(3) | AAR | Postoperative and discharge |  |  |  |  |  |
| C-31 | Type B | M，40-45 | H+S | / | TAMTI | Postoperative and discharge |  |  |  |  |  |
| C-38 | Type A | M，30-35 | / | BT+LTTA+LSA | AAR+TAR+SET | Postoperative and discharge |  |  |  |  |  |
| C-39 | Type B | M，66-70 | / | / | TAMTI | Postoperative and discharge |  |  |  |  |  |
| C-40 | Type A | M，46-50 | H+S | BT+LSA+PCE | CSA+AAR+TAR+SET | Postoperative and discharge |  |  |  |  |  |
| C-42 | Type A | F，60-65 | H | / | / | Dead（Patient rejected surgery） |  |  |  |  |  |
| C-43 | Type A | M，60-65 | S | CAHD | TAMTI | Postoperative and discharge |  |  |  |  |  |
| C-44 | Type A | F，50-55 | H | BT+LCA+RCA+PCE+AR(2) | AAR+TAR+SET | Postoperative and discharge |  |  |  |  |  |
| C-45 | Type A | M，50-55 | / | LSA+PCE | AAR+TAR+SET | Postoperative and discharge |  |  |  |  |  |
| C-46 | Type A | F，46-50 | H | BT+LTTA+LSA+AR(3) | BT+TAR+SET | Postoperative and discharge |  |  |  |  |  |
| C-48 | Type B | F，50-55 | H | / | TAMTI | Postoperative and discharge |  |  |  |  |  |
| C-49 | Type A | M，56-60 | H | / | / | Dead before surgery |  |  |  |  |  |
| C-50 | Type A | M，40-45 | S | BT+LTTA+LSA+PCE | NCSA+AAR+TAR+SET | Postoperative and discharge |  |  |  |  |  |
| C-51 | Type A | M，36-40 | S | / | TAMTI | Postoperative and discharge |  |  |  |  |  |
| C-52 | Type B | M，36-40 | H | BT+LTTA+LSA+LCA+RCA+PCE+AR(1) | BT+TAR+SET | Postoperative and discharge |  |  |  |  |  |
| C-53 | Type A | M，66-70 | H | BT+LTTA+LSA+RCA | CSA+AAR+TAR+SET+CABG | Dead after surgery |  |  |  |  |  |
| C-54 | Type A | M，30-35 | / | BT | AAR+TAR+SET | Postoperative and discharge |  |  |  |  |  |
| C-55 | Type A | M，50-55 | / | BT+LTTA+LSA+PCE+AR(2) | NCSA+AAR+TAR+SET | Postoperative and discharge |  |  |  |  |  |
| C-57 | Type A | F，40-45 | H | PCE | AAR+TAR+SET | Postoperative and discharge |  |  |  |  |  |
| C-58 | Type A | M，60-65 | H+S | BT+LTTA+LSA+PCE+AR(2) | NCSA+AAR+TAR+SET | Postoperative and discharge |  |  |  |  |  |
| C-61 | Type B | F，46-50 | H | / | / | Postoperative and discharge |  |  |  |  |  |
| C-64 | Type A | M，46-50 | H | PCE | AAR+TAR+SET | Postoperative and discharge |  |  |  |  |  |
| C-65 | Type A | M，36-40 | H+S | / | / | Patient rejected surgery and diacharged |  |  |  |  |  |
| C-66 | Type A | M，56-50 | S | / | / | Dead（Patient rejected surgery） |  |  |  |  |  |
| C-68 | Type B | F，60-65 | / | / | / | Discharge（Patient rejected surgery） |  |  |  |  |  |
| C-69 | Type B | F，50-55 | H | / | / | Postoperative and discharge |  |  |  |  |  |
| C-70 | Type A | M，50-55 | H+S | BT+LTTA+LSA+PCE+CAHD | AAR+TAR+SET | Dead before surgery |  |  |  |  |  |
| C-73 | Type A | M，50-55 | S | PCE+AR(1) | AAR+TAR+SET | Postoperative and discharge |  |  |  |  |  |
| C-74 | Type A | M，40-45 | / | PCE+AR(1) | AAR+TAR+SET | Postoperative and discharge |  |  |  |  |  |
| C-76 | Type B | M，50-55 | H | / | / | Patient rejected surgery and diacharged |  |  |  |  |  |
| C-77 | Type B | M，50-55 | / | / | TAMTI | Postoperative and discharge |  |  |  |  |  |
| C-79 | Type A | F，50-55 | H | BT+LTTA+LSA+PCE | AAR+TAR+SET | Postoperative and discharge |  |  |  |  |  |
| C-80 | Type B | M，60-65 | H | / | TAMTI | Postoperative and discharge |  |  |  |  |  |
| C-81 | Type A | F，60-65 | / | PCE | / | Dead（Patient rejected surgery） |  |  |  |  |  |
| C-82 | Type B | M，46-50 | H+S | / | TAMTI | Postoperative and discharge |  |  |  |  |  |
| C-83 | Type A | M，36-40 | S | / | / | Dead（Patient rejected surgery） |  |  |  |  |  |
| C-85 | Type B | M，50-55 | / | / | TAMTI | Postoperative and discharge |  |  |  |  |  |
| C-86 | Type A | M，56-60 | H+S | BT+RCA+AR(2) | BT+TAR+SET | Postoperative and discharge |  |  |  |  |  |
| C-87 | Type A | M，40-45 | S | BT+PCE+AR(3)+MR(2)+TR(2) | BT+TAR+SET | Postoperative and discharge |  |  |  |  |  |
| C-90 | Type A | M，36-40 | H | / | TAMTI | Postoperative and discharge |  |  |  |  |  |
| C-91 | Type A | M，26-30 | / | BT+PCE+MR(1) | AAR+TAR | Postoperative and discharge |  |  |  |  |  |
| C-94 | Type B | M，56-60 | H | / | TAMTI | Postoperative and discharge |  |  |  |  |  |
| C-98 | Type B | M，40-45 | S | / | TAMTI | Postoperative and discharge |  |  |  |  |  |
| C-100 | Type A | M，40-45 | / | / | TAMTI | Postoperative and discharge |  |  |  |  |  |
| C-103 | Type B | F，56-60 | H | / | TAMTI | Postoperative and discharge |  |  |  |  |  |
| C-104 | Type B | M，40-45 | H | / | TAMTI | Postoperative and discharge |  |  |  |  |  |
| C-105 | Type B | M，56-60 | H | / | TAMTI | Postoperative and discharge |  |  |  |  |  |
| C-110 | Type B | F，20-25 | PIH | BT | TAMTI | Postoperative and discharge |  |  |  |  |  |
| C-111 | Type B | F，56-60 | H | / | TAMTI | Postoperative and discharge |  |  |  |  |  |
| C-114 | Type A | M，50-55 | / | BT+LTTA+LSA | TAMTI | Postoperative and discharge |  |  |  |  |  |
| C-116 | Type B | M，50-55 | H | / | TAMTI | Postoperative and discharge |  |  |  |  |  |
| C-117 | Type B | F，70-75 | H | / | / | Discharge（Patient rejected surgery） |  |  |  |  |  |
| C-119 | Type B | M，60-65 | / | / | TAMTI | Postoperative and discharge |  |  |  |  |  |
| C-120 | Type A | M，40-45 | H | LTTA+LSA | TAMTI | Postoperative and discharge |  |  |  |  |  |
| C-121 | Type B | M，66-70 | H | / | TAMTI | Postoperative and discharge |  |  |  |  |  |
| C-122 | Type B | M，36-40 | H | PCE | TAMTI | Postoperative and discharge |  |  |  |  |  |
| C-123 | Type A | M，60-65 | H+S | PCE | TAMTI | Postoperative and discharge |  |  |  |  |  |
| C-124 | Type B | F，56-60 | H | / | TAMTI | Postoperative and discharge |  |  |  |  |  |
|  |  |  |  |  |  |  |  |  |  |  |  |
| **AR:** aortic regurgitation(1 mild, 2 moderate, 3 severe); **CAHD**: coronary atherosclerotic heart disease; **CVS**: cardiovascular system; **M**:male; **MR**: mitral regurgitation(1 mild, 2 moderate, 3 severe); **F**: female; **H**: hypertension; **S**: smoking; **HC**: hemopericardium; **PIH**: pregnancy-induced hypertension syndrome; **PCE**: pericardial effusion; **TR**: tricuspid valve regurgitation(1 mild, 2 moderate, 3 severe); **BT**: brachiocephalic trunk involvement of the dissection; **LCCA**: left common carotid artery involvement of the dissection; **LSA**: left subclavian artery involvement of the dissection; **RCA**: right coronary artery involvement of the dissection; **LCA**: left coronary artery involvement of the dissection; **AAR**: ascending aorta replacement; **TAR**: total arch replacement; **CABG**: coronary artery bypass graft; **NCSA**: no coronary sinus angioplasty; **CSA**: coronary sinus angioplasty; **SET**: stented elephant trunk; **BT**: bentall; **TAMTI**: thoracic aortic membrane-covert stent implantation; **LCSA**: left coronary sinus angioplasty. | | | | | | | | | | | |
|  |  |  |  |  |  |  |  |  |  |  |  |
|  |  |  |  |  |  |  |  |  |  |  |  |
|  |  |  |  |  |  |  |  |  |  |  |  |
|  |  |  |  |  |  |  |  |  |  |  |  |
|  |  |  |  |  |  |  |  |  |  |  |  |
|  |  |  |  |  |  |  |  |  |  |  |  |
|  |  |  |  |  |  |  |  |  |  |  |  |
